# Enhanced anti-tumor efficacy of tumor-infiltrating lymphocytes by GITR agonist in ovarian cancer

**DOI:** 10.3389/fimmu.2025.1670841

**Published:** 2025-11-06

**Authors:** Daun Jung, Ah-Ra Goh, Ki Yeon Kim, Ji Min Lee, Eun Ji Lee, Sohyun Hwang, Haeyoun Kang, Hyun Park, Hee Jung An

**Affiliations:** 1 CHA Biomedical Research Institute, CHA Bundang Medical Center, CHA University School of Medicine, Seongnam, Republic of Korea; 2 CHA Advanced Research Institute, CHA Bundang Medical Center, Seongnam, Republic of Korea; 3 Department of Pathology, CHA Bundang Medical Center, CHA University, Seongnam, Republic of Korea; 4 Department of Gynecologic Oncology, CHA Bundang Medical Center, CHA University, Seongnam, Republic of Korea

**Keywords:** tumor-infiltrating lymphocytes, T cell expansion, GITR, ovarian cancer, cancer immunotherapy, PDCX

## Abstract

**Background:**

Adoptive cell therapy (ACT) using autologous tumor-infiltrating lymphocytes (TILs) is a personalized immunotherapy that has shown promising clinical results in various tumor types. Although TILs are associated with improved survival in patients with ovarian cancer (OC), their therapeutic efficacy remains limited. Therefore, novel strategies to enhance the anti-tumor activity of TILs are needed to improve outcomes in OC treatment.

**Methods:**

Single cells were isolated from tumor tissues of patients with high-grade serous carcinoma (HGSC) and expanded for 14 days in the presence of IL-2 under four different conditions: (1) control (W), (2) PD-1 antagonist (WI), (3) PD-1 antagonist + IL-15 + IL-21 (WIO), and (4) PD-1 antagonist + IL-15 + IL-21 + GITR-agonist (WIOG). Following validation of TIL purity and activation phenotypes by flow cytometry, RNA sequencing was performed to elucidate the underlying mechanisms. *In vitro* efficacy was assessed using a 7-AAD/Far-Red cytotoxicity assay against autologous tumor cells, and *in vivo* efficacy was evaluated in NSG mice bearing subcutaneous patient-derived tumor cell xenografts (PDCX).

**Results:**

On day 14, the WIOG group showed a 1.3-fold increase in expansion compared to the control group, along with a high CD8^+^/Treg ratio (454.6). Furthermore, both CD8^+^ and CD4^+^ T cells in the WIOG group exhibited elevated Granzyme B expression. RNA sequencing identified 279 upregulated genes associated with T cell activation (*CSF2, TNFRSF4*), cytotoxicity (*IFNG, GZMB*), and anti-apoptosis (*BMF, BCL2L1*). Compared to the controls, the WIOG group demonstrated a 1.9-fold increase in cytolytic activity *in vitro* and a 56% reduction in tumor growth in the patient-derived tumor cell xenograft (PDCX) model.

**Conclusions:**

Taken together, we demonstrated that the addition of an agonistic GITR antibody during the early phase of TIL culture increased the CD8^+^ T cell to Treg cell ratio and enhanced anti-tumor T cell immunity. Enhancing TILs with a GITR agonist may be beneficial for improving the clinical outcomes of TIL-based ACT in OC.

## 1 Introduction

Ovarian cancer (OC), especially high-grade serous carcinoma (HGSC) type, is one of the most lethal gynecologic malignancies, characterized by a poor prognosis and a very high mortality rate. Approximately 70-75% of patients are diagnosed at an advanced stage (stage III or IV) due to the lack of early diagnosis (1). The standard treatment involves surgical resection followed by chemotherapy; however, over 80% of patients with advanced-stage disease experience recurrence within 2–3 years of first-line therapy (2), resulting in a 5-year survival rate of only 17% (3). These statistics highlight a critical need for the development of novel and effective therapeutic strategies.

The field of cancer immunotherapy has advanced remarkably over the past decade (4). Among the various strategies, adoptive cell therapy (ACT) has emerged as one of the most potent approaches. The three principal forms of ACT include chimeric antigen receptor (CAR) T-cell therapy, engineered T-cell receptor (TCR) T-cell therapy, and tumor-infiltrating lymphocyte (TIL) therapy. Both CAR-T and TCR-T therapies involve genetically modifying a patient’s T cells to recognize and target tumor-associated antigens (5–7), therefore they carry the risk of off-target effects, potentially leading to toxicity due to the recognition of antigens expressed on normal tissues. Moreover, while CAR-T cells have demonstrated remarkable success in treating hematologic malignancies (8), their effectiveness against solid tumors remains limited. TCR-T therapy, on the other hand, holds promise for targeting solid tumors by recognizing specific antigens, including intracellular ones. However, this approach is also limited by major histocompatibility complex (MHC) restriction and its specificity to a single antigen.

In contrast, TILs offer natural tumor specificity without the need for genetic engineering, possess broad antigen recognition capabilities, and have demonstrated potential in the treatment of solid tumors, where other therapies have faced significant limitations. Notably, ACT with TILs has shown high efficacy in the treatment of metastatic melanoma (MM) (9–11). Promising clinical outcomes have also been reported in other malignancies, including cervical squamous cell carcinoma (12), cholangiocarcinoma, non-small cell lung cancer (13), colorectal cancer (14), and breast cancer (15, 16). Furthermore, lifileucel (Amtagvi; Iovance Biotherapeutics, Inc.), a TIL-based immunotherapy, was recently approved by the U.S. Food and Drug Administration (FDA) for the treatment of advanced melanoma (17). However, clinical trials of TIL therapy in other solid tumors have yielded mixed and sometimes inconclusive results.

A possible explanation for the limited success of TIL therapy in certain tumors is the functional exhaustion of TILs within the tumor microenvironment or during the ex vivo expansion process, which diminishes their cytotoxic efficacy. A key contributor to TIL dysfunction is the upregulation of immune checkpoint molecules such as programmed cell death protein 1 (PD-1), which impair T-cell activity and reduce their ability to eliminate cancer cells (18). Consequently, combining TIL therapy with immune checkpoint inhibitors has emerged as a promising strategy to reinvigorate TIL function and restore their tumor-killing potential (19, 20). In addition, TIL activity and proliferation can be further enhanced through the use of cytokine combinations, particularly interleukin-2 (IL-2), interleukin-15 (IL-15), and interleukin-21 (IL-21), which promote T-cell expansion and survival Among these, IL-15 and IL-21 are gaining attention in cancer immunotherapy due to their pivotal roles in supporting the *ex vivo* expansion of TILs (21–23). These cytokines enhance T-cell proliferation, longevity, and effector functions, thereby increasing their anti-tumor capacity (24–26).

In addition, we utilized an agonistic anti-GITR antibody to enhance the therapeutic efficacy of TILs in this study. Glucocorticoid-induced TNF receptor family-related protein (GITR) is a co-stimulatory receptor that plays a pivotal role in modulating T cell effector functions. GITR is predominantly expressed on regulatory T cells (Tregs) and activated effector T cells. Engagement of GITR signaling via agonistic antibodies promotes the proliferation and activation of effector T cells while concurrently diminishing the suppressive activity of Tregs. This dual mechanism amplifies the overall anti-tumor immune response (27–30). Based on these findings, we aimed to augment the anti-tumor capacity of TILs by incorporating an anti-GITR antibody into the culture protocol.

Building on recent advances in enhancing T cell functionality, we cultured TILs derived from HGSC tumors under various combinations of IL-15/21 cytokines, an antagonistic anti-PD-1 antibody, and an agonistic anti-GITR antibody to improve their activity and expansion for the development of effective TIL-based cell therapy. This study demonstrates that incorporating an agonistic anti-GITR antibody during the early phase of TIL culture increases the proportion of CD8^+^ T cells and enhances their functional capacity. Furthermore, we report, for the first time, the potent anti-tumor efficacy of anti-GITR–augmented TILs against primary cancer cells, both *in vitro* and *in vivo.*

## 2 Materials and methods

### 2.1 Patient information and tumor processing

We evaluated tumor samples from ten chemotherapy-naive patients with high-grade serous carcinomas (HGSCs) at various cancer stages, as detailed in **Supplementary Table S1**. This study was conducted in accordance with the Declaration of Helsinki and was approved by the Institutional Review Board of CHA University, CHA Bundang Medical Center (IRB No. 2019-08-039). All patients provided written informed consent for specimen collection.

Tumor tissues were dissociated into single-cell suspensions using the MACS Human Tumor Dissociation Kit (Miltenyi Biotec, Bergisch Gladbach, Germany; #130-095-929) according to the manufacturer’s instructions. Briefly, tumor tissues were cut into 2–4 mm³ fragments and enzymatically digested using the enzyme mix provided in the kit, in combination with the h_tumor_02 program on a gentleMACS Dissociator and C tubes (Miltenyi Biotec). The dissociation was further processed using the h_tumor_03 program on the gentleMACS tissue Dissociator (Miltenyi Biotec; #130-093-235).

The enzymatic reaction was quenched using a medium containing antibiotic-antimycotic (AA; Gibco/Thermo Fisher Scientific, Waltham, MA, USA; #15240062) and fetal bovine serum (FBS; Gibco; #26140079). The resulting suspension was filtered through a 70-μm cell strainer (BD Falcon, Franklin Lakes, NJ, USA; #352350) and layered onto Ficoll-Paque PLUS (Cytiva; #17144002) to isolate mononuclear cells. The collected single-cell pellets were washed and either resuspended in TIL culture medium or preserved in cell-freezing medium for cryostorage in liquid nitrogen.

### 2.2 Primary cancer cell culture

Autologous primary ovarian cancer cultures were established in parallel with TIL cultures. Following tumor dissociation, a portion of the cell suspension in Ficoll solution was centrifuged at 90 × g for 2 minutes. The resulting cell pellet was resuspended in McCoy’s 5A medium (Gibco; #16600082) supplemented with 10% fetal bovine serum (FBS), 1% antibiotic-antimycotic (AA), and 20 ng/mL epidermal growth factor (EGF; Gibco; #PHG0311), and seeded into a T75 flask (SPL Life Sciences, Pocheon, South Korea; #70075). Primary cultures were passaged upon reaching approximately 80% confluency. To confirm that the primary cultures represented epithelial ovarian cancer cells, they were characterized by flow cytometry.

### 2.3 Human cell lines

The human leukemia cell line K562 was purchased from the American Type Culture Collection (ATCC, Manassas, VA, USA) and cultured according to the ATCC’s recommended conditions. The A2780cis ovarian cancer cell line (RRID: CVCL_0134) was obtained from the European Collection of Authenticated Cell Cultures (ECACC) and maintained under the conditions recommended by ECACC.

### 2.4 TIL expansion

Following tumor processing, single cells were cultured in complete medium (CM) for 14 days. The CM used for TIL culture consisted of a 1:1 mixture of RPMI 1640 (Gibco; #72400-047) supplemented with 10% human serum albumin (Sigma-Aldrich, St. Louis, MO, USA; #H4522), 1% antibiotic-antimycotic (AA), 55 µM 2-mercaptoethanol (Gibco; #21985023), and AIM-V medium (Gibco; #12055-091).

TILs were expanded under four IL-2–containing conditions (PeproTech; #200-02) (1): control (W group) (2), anti-PD1 antibody (BioXcell, #BE0188, WI group) (3), anti-PD1 antibody + IL-15 + IL-21 (WIO group), and (4) anti-PD1 antibody + IL-15 + IL-21 +anti-GITR antibody (BPS Bioscience, San Diego, CA, USA, #79053-2, WIOG group). Cultures were either split or scaled up to maintain less than 80% confluency, or half of the medium was replaced every 2–3 days.

For the *in vivo* efficacy study, TILs were expanded using a rapid expansion protocol (REP) for 9 days with an anti-CD3/CD28/CD2 T cell activator (STEMCELL Technologies, Vancouver, BC, Canada; #10970). A total of 1 × 10^7^ TILs were seeded in 10 mL of CM containing 3000 IU/mL IL-2 and the T cell activator. On day 1, an additional 10 mL of CM supplemented with 3000 IU/mL IL-2 was added. Starting on day 2, half of the culture medium was replaced daily. From day 5 onward, cultures were either transferred to GREX 6-well plates (Wilson Wolf Manufacturing, Saint Paul, MN, USA; #80240M) or scaled up to GREX-100M (Wilson Wolf Manufacturing) to maintain less than 80% confluency. The final concentrations of cytokines and antibodies used in each condition are listed in **Supplementary Table S2**.

### 2.5 Flow cytometry

Cells were stained with antibodies listed in **Supplementary Table S3** by incubating them in the dark at 4 °C for 30 minutes. For intracellular staining, anti-FOXP3 or anti-Granzyme B antibodies (both from eBioscience, San Diego, CA, USA) were used following fixation and permeabilization with BD CytoFix/CytoPerm™ solution (BD Biosciences; #554714). Stained cells were analyzed using a CytoFLEX flow cytometer (Beckman Coulter, Brea, CA, USA), and data were processed with FlowJo software (version 10.1; Treestar Inc., Ashland, OR, USA; RRID: SCR_008520).

### 2.6 RNA sequencing

After staining with anti-CD3, CD4, and CD8 antibodies followed by washing, CD8^+^ T cells were sorted using a MoFlo XDP High-Speed Cell Sorter (Beckman Coulter). RNA was extracted from sorted cells at days 0 and 14 using TRIzol reagent (Invitrogen, Carlsbad, CA, USA; #15596026). RNA integrity was assessed using the TapeStation RNA ScreenTape system (Agilent Technologies, Santa Clara, CA, USA). For cDNA library preparation, the TruSeq Stranded mRNA LT Sample Prep Kit (Illumina Inc., San Diego, CA, USA) was used. The library preparation protocol included poly(A)-selected RNA extraction, RNA fragmentation, reverse transcription with random hexamer primers, and 100-nt paired-end sequencing on an Illumina NovaSeq 6000 platform. Transcript assembly was performed using StringTie v1.3.4d (Pertea et al., 2015, 2016), and expression profiles were used for downstream analyses, including differential gene expression (DEG) analysis. Gene classification and Gene Ontology (GO) enrichment analyses were conducted using DAVID (http://david.abcc.ncifcrf.gov/; RRID: SCR_001881) and Enrichr (https://maayanlab.cloud/Enrichr/). RNA-seq data supporting the findings of this study have been deposited in the Gene Expression Omnibus (GEO; RRID: SCR_005012) under the accession number GSE278940.

### 2.7 Cytotoxicity assay

TIL cytotoxicity against autologous primary cancer cells was evaluated using a Far Red/7-aminoactinomycin D (7-AAD) flow cytometry–based assay. Target cells were first stained with Far Red dye (Thermo Fisher Scientific; #C34564), then co-cultured with TILs in a 96-well plate for 20 hours at a defined effector-to-target (E:T) ratio. After incubation, cells were resuspended in PBS containing 7-AAD (Invitrogen; #00-6993-50), and target cell lysis was assessed using a CytoFLEX flow cytometer and analyzed with FlowJo software.

In addition, a microscopic cytotoxicity assay was performed using calcein (AAT Bioquest; #21905) and Far Red staining, and visualized by fluorescence microscopy to assess cell viability and lysis.

### 2.8 Establishment of the patient-derived cancer cell xenograft model

We have previously established a Patient-Derived Cancer cell Xenograft (PDCX) model using cryopreserved tumor cells (31), and a brief description of the method is provided below.

Cryopreserved single cells derived from human tumors were thawed, and the cell-freezing medium was removed. Patient-derived tumor cells (4 × 10^6^) were mixed with an equal volume of Matrigel (Corning Incorporated, Corning, NY, USA; #354248) and subcutaneously injected into the flanks of 7–8-week-old female NSG mice (JAbio, Suwon, Republic of Korea) to generate F1 tumors. F1 tumors exceeding 500 mm³ in volume were excised and enzymatically dissociated using HBSS (Gibco; #14175-095) supplemented with 2 mg/mL Collagenase/Dispase (Roche, Basel, Switzerland; #10103578001) and 70 U/mL DNase I (Sigma-Aldrich; #D5025) at 37 °C for 20 minutes. The digested tissue was then filtered through a 70-µm cell strainer (SPL Life Sciences; #93070).

The resulting single-cell suspension was washed and resuspended in a cell-freezing medium composed of a 5:4:1 mixture of complete medium (as described in the primary cancer cell culture section), FBS (Gibco), and DMSO (Sigma-Aldrich; #D4540), and subsequently cryopreserved in liquid nitrogen. Cryopreserved single cells from F1 tumors were later passaged using the same protocol to generate F2 tumors.

### 2.9 *In vivo* anti-tumor efficacy in PDCX model

Xenograft tumors were established in 7- to 8-week-old female NSG mice (NOD.Cg-Prkdcscid Il2rgtm1Wjl/SzJ; JAbio). Mice were housed under standard, specific pathogen-free (SPF) conditions at the Laboratory Animal Research Center of CHA University. All animal experiments were reviewed and approved by the Institutional Animal Care and Use Committee of CHA University (IACUC No. 240045) and conducted in accordance with the ARRIVE guidelines.

Cryopreserved F2 tumor cells (4 × 10^6^) were thawed and subcutaneously injected into the right flank of each mouse. Tumor volumes were measured regularly using calipers and calculated using the formula: volume = (width² × length)/2. Nine days after implantation, tumor growth was confirmed, and mice were randomized based on tumor volume into vehicle control and WIOG TIL treatment groups (n = 5 mice per group). Group allocation, treatment, and monitoring were conducted in a blinded and randomized manner to minimize potential confounding variables.

WIOG TILs (1 × 10^7^) were administered intravenously on days 9 and 28. Recombinant human IL-2 (45,000 IU/mouse; PeproTech) was administered daily by subcutaneous injection for 16 consecutive days following TIL infusion. Tumor volume was measured three times per week, and all mice were euthanized on day 45.

For analysis of immune cell persistence, tumors were excised and dissociated into single-cell suspensions. Cells were stained and washed as described previously and analyzed by flow cytometry using the antibodies listed in **Supplementary Table S4**. Sample size was determined based on *a priori* power analysis using estimated effect size and standard deviation. All animals and data points were included in the analysis; no animals or experimental units were excluded.

### 2.10 Statistical analysis

Data are presented as mean ± standard deviation (SD). Statistical analyses were performed using the Wilcoxon signed-rank test or the Mann–Whitney U test with GraphPad Prism software (version 10; GraphPad, La Jolla, CA, USA; RRID: SCR_002798). A *P*-value of less than 0.05 was considered statistically significant. Statistical significance is indicated as follows: *P < 0.05, **P < 0.01, ***P < 0.001, and ****P< 0.0001.

## 3 Results

### 3.1 Anti-GITR enhances ovarian TIL expansion by promoting CD8^+^ T cell proliferation and suppressing Tregs

TILs were generated from single cells obtained through enzymatic and mechanical dissociation of freshly resected tumors, rather than from tumor fragments. This approach was based on preliminary findings showing that single cell–derived TILs exhibit greater expansion and cytotoxicity than fragment-derived TILs (**Supplementary Figure S1**).

**Figure 1:**
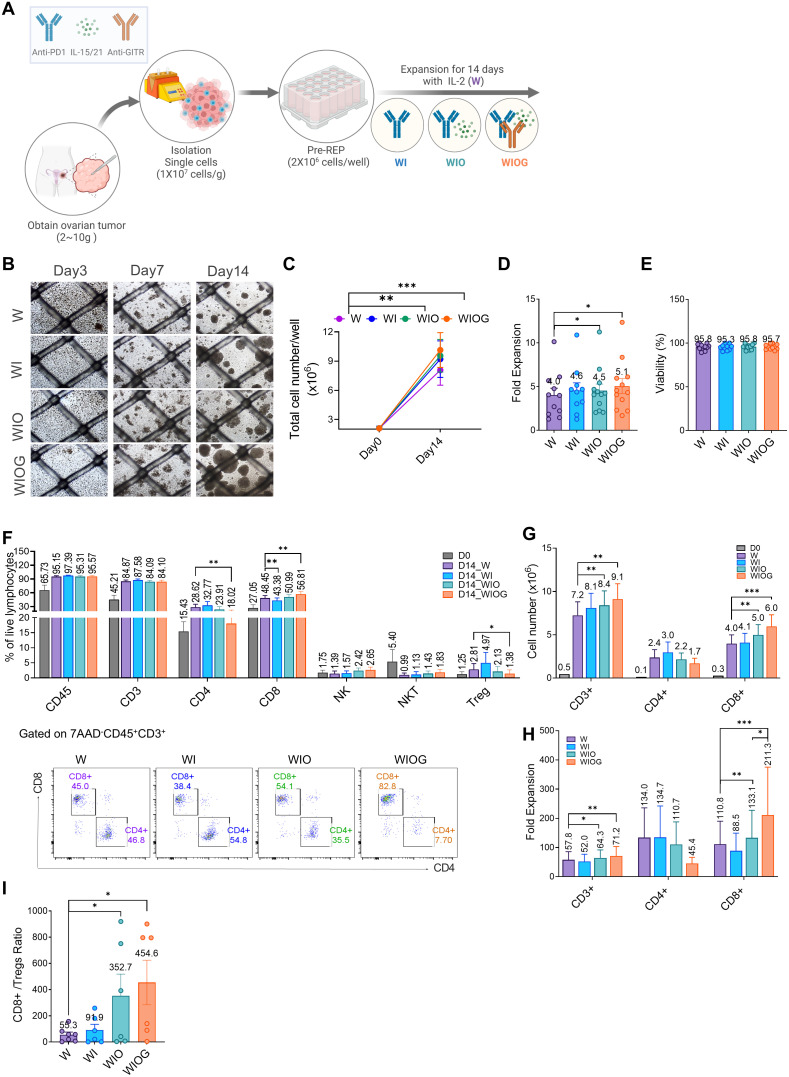
Anti-GITR increased CD8+ T cells and decreased Treg cells. **(A)** Schematic illustration of TIL expansion from ovarian cancer tissue. HGSC samples were obtained, dissociated into single-cell suspension, and expanded for 14 days with IL-2 under four different conditions: W (IL-2 alone), WI (IL-2 + Anti-PD1), WIO (IL-2 + Anti-PD1 + IL-15/21), and WIOG (IL-2 + Anti-PD1 + IL-15/21 + Anti-GITR). **(B)** Representative images of cell growth in each condition at days 3, 7, and 14. **(C)** Total cell number on days 0 and 14 under each condition. **(D)** Fold expansion of TILs expanded in each condition at day 14. **(E)** Viability of TILs at day 14 across different conditions. **(F)**Top: Percentages of CD45^+^, CD3^+^, CD4^+^ T cells, CD8^+^ T cells, NK cells, NKT cells, and Treg cells were analyzed using flow cytometry. Bottom: Representative plots of CD4^+^ and CD8^+^ T cells from each condition are shown after gating on 7AAD^-^CD45^+^CD3^+^ cells. The absolute cell numbers **(G)** and fold expansion **(H)** of CD3^+^, CD4^+^ and CD8^+^ T cells in each condition at day 14, as well as the ratio of CD8^+^/Tregs **(I)** were calculated. Data are presented as mean ± SD (n=10). Statistical analyses were performed using the Wilcoxon-test (*P < 0.05, **P < 0.01, ***P < 0.001).

To enhance ovarian TIL expansion and anti-tumor activity, we cultured TILs with single cells from fresh ovarian tumors under four different conditions (1):W (IL-2 alone), (2)WI (IL-2 + anti-PD1), (3)WIO (IL-2 + anti-PD1 + IL-15/21), and (4)WIOG (IL-2 + anti-PD1 + IL-15/21 + anti-GITR) (**Figure 1A**).

Among the four groups, the WIOG condition exhibited the highest TIL expansion, with a 5.1-fold increase on day 14 and the formation of the largest cell aggregates, enhanced proliferation induced by GITR agonism (**Figures 1B–D**).

TILs cultured under all four conditions showed high viability (95–97%) (**Figure 1E**). The proportions of CD45^+^ and CD3^+^ cells increased from 65.7% and 45.2% on day 0 to 95.2–97.4% and 84.1–87.6%, respectively, on day 14 across all four groups (**Figure 1F**). While the proportions of CD3^+^ and CD8^+^ cells were comparable across groups, the absolute numbers and fold expansion of CD3^+^ and CD8^+^ cells were significantly higher in the WIO and WIOG groups compared to the W group (**Figures 1G, H**).

In particular, the WIOG group showed a significant increase in the expansion of CD8+ T cells based on Day 0 (211-fold vs. 110-fold; **Figure 1H**), along with a significant decrease in the proportion of CD4^+^ T cells and regulatory T cells (Tregs) compared to the W group (1.38% vs. 2.81%; **Figure 1F**). Consistent with the reduction in FOXP3^+^CD4^+^ Tregs, the proportion of FOXP3^+^ CD8^+^ Tregs was also lower in the WIOG group compared to the W group (0.48% vs. 1.01%) (**Supplementary Figure S2**).

Although the WIO group showed a similar trend toward reduced CD4^+^ and increased CD8^+^ T cell proportions compared to the W group, these changes were not statistically significant and were less pronounced than those observed in the WIOG group. The CD8^+^ T cell to Treg (CD8^+^/Treg) ratio was markedly elevated in both the WIO (352.7) and WIOG (454.6) groups (**Figure 1I**). These results suggest that anti-GITR promotes the expansion of cytotoxic CD8^+^ T cells while concurrently suppressing Tregs.

### 3.2 Anti-GITR stimulation increases the proportion of Granzyme B^+^ T cells within expanded TILs

**Figure 2:**
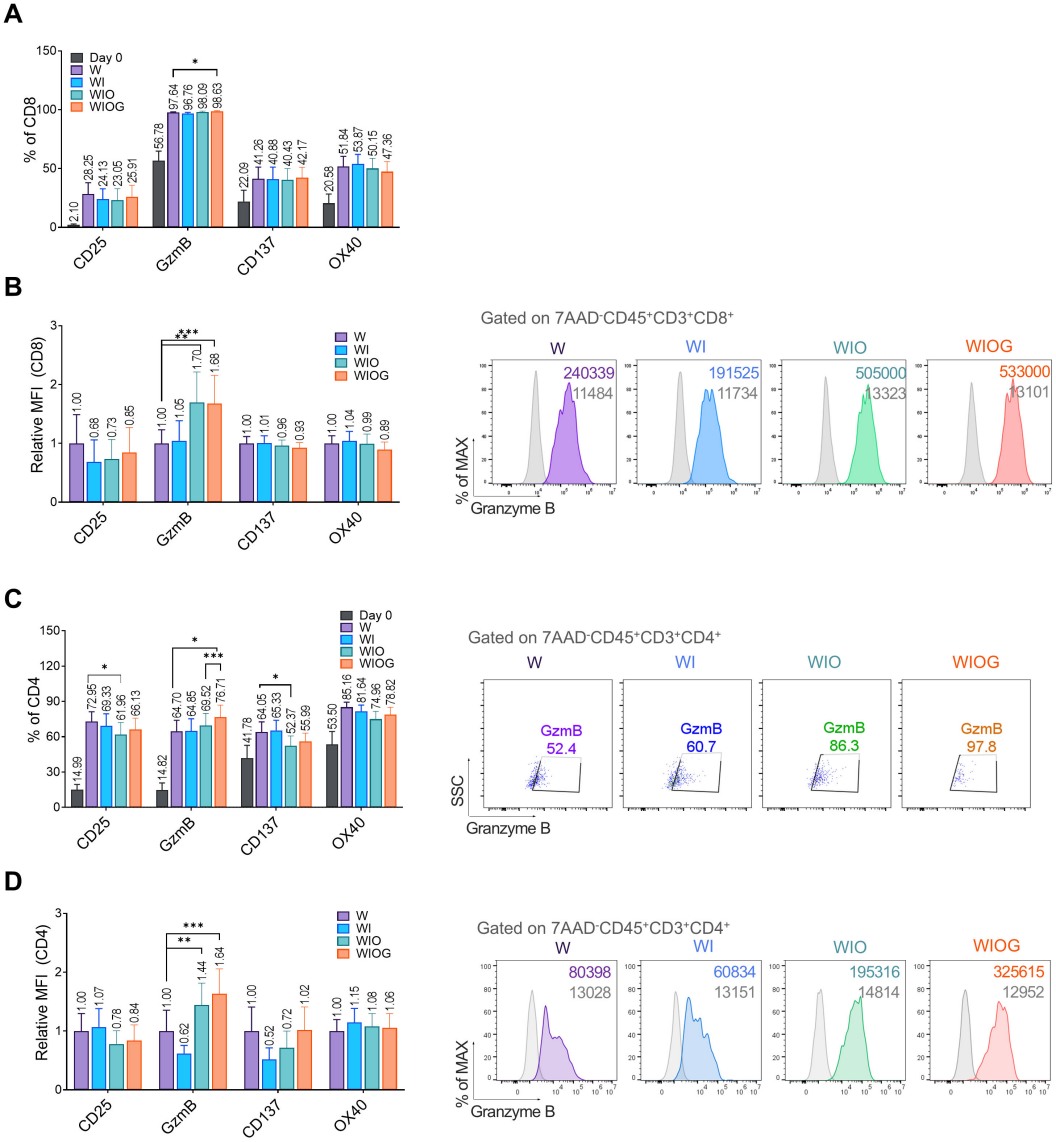
Phenotypic analysis of expanded TILs. **(A)** Percentages of CD8^+^ T cells expressing activation markers CD25, Granzyme B (GzmB), CD137, and OX40 under four different expansion conditions (W, WI, WIO, and WIOG) and at Day 0 **(B)** Left: Relative median fluorescence intensity (MFI) of activation markers on CD8^+^ T cells, measured by flow cytometry. Right: Representative histograms of GzmB expression in CD8^+^ T cells gated on 7AAD^-^CD45^+^CD3^+^CD8^+^ populations across the four conditions; grey histograms represent isotype controls. **(C)** Left: Percentage of CD4^+^ T cells expressing activation markers CD25, GzmB, CD137 and OX40 under four different expansion conditions. Right: Representative dot plots showing GzmB^+^ expression in CD4^+^ T cells gated on 7AAD^-^CD45^+^CD3^+^CD4^+^ populations under each condition. **(D)** Left: Relative MFI of activation markers on CD4^+^ T cells, determined by flow cytometry. Right: Representative histograms of GzmB expression in CD4^+^ T cells gated on 7AAD^-^CD45^+^CD3^+^CD4^+^ populations under the four conditions, with grey histograms indicating isotype controls. Data are presented as mean ± SD (n=10). Statistical analyses were performed using the Wilcoxon-test (*P < 0.05, **P < 0.01, ***P < 0.001).

We evaluated the expression of activation markers on both CD8^+^ and CD4^+^ T cells within expanded TILs using flow cytometry. Compared to day 0, both CD8^+^ and CD4^+^ T cells in the expanded TILs showed significant increases in the percentages of CD25^+^ and granzyme B (GzmB^+^) cells. The expression of OX40 and 4-1BB was also slightly elevated (**Figures 2A, C**).

In CD8^+^ T cells, all groups exhibited a high proportion of GzmB^+^ cells (96–98%) (**Figure 2A**). Notably, the relative mean fluorescence intensity (MFI) of GzmB was significantly higher in the WIO and WIOG groups compared to the W group (**Figure 2B**), suggesting increased granzyme B expression at the single-cell level. In contrast, the expression levels of CD25, CD137, and OX40 did not differ significantly among the groups (**Figures 2A, B**).

In CD4^+^T cells, the proportion of GzmB^+^ cells was significantly increased in the WIOG group (76.7%) compared to the W (64.7%, *P* = 0.0161) and WIO (69.5%, *P* = 0.0010) groups (**Figure 2C**). Consistent with the results of CD8^+^ T cells, the relative MFI of GzmB^+^ in CD4^+^ T cells was also significantly elevated in the WIOG group compared to the W group (**Figures 2C, D**).

Memory phenotype analysis revealed that the majority of CD4^+^ and CD8^+^ T cells in expanded TILs exhibited an effector memory (EM) phenotype. Although the WIOG group showed a slight increase in the frequency of EMRA (effector memory RA^+^) cells compared to day 0, the difference was not statistically significant and was lower than that observed in the WIO group (Supplementary **Figures 3A, B**). Immune checkpoint molecules such as PD-1 (36–38%), CTLA-4 (43–48%), and TIM-3 (25–31%) were moderately expressed on both CD8^+^ and CD4^+^ T cells across all groups, with no significant differences between groups (data not shown).

**Figure 3:**
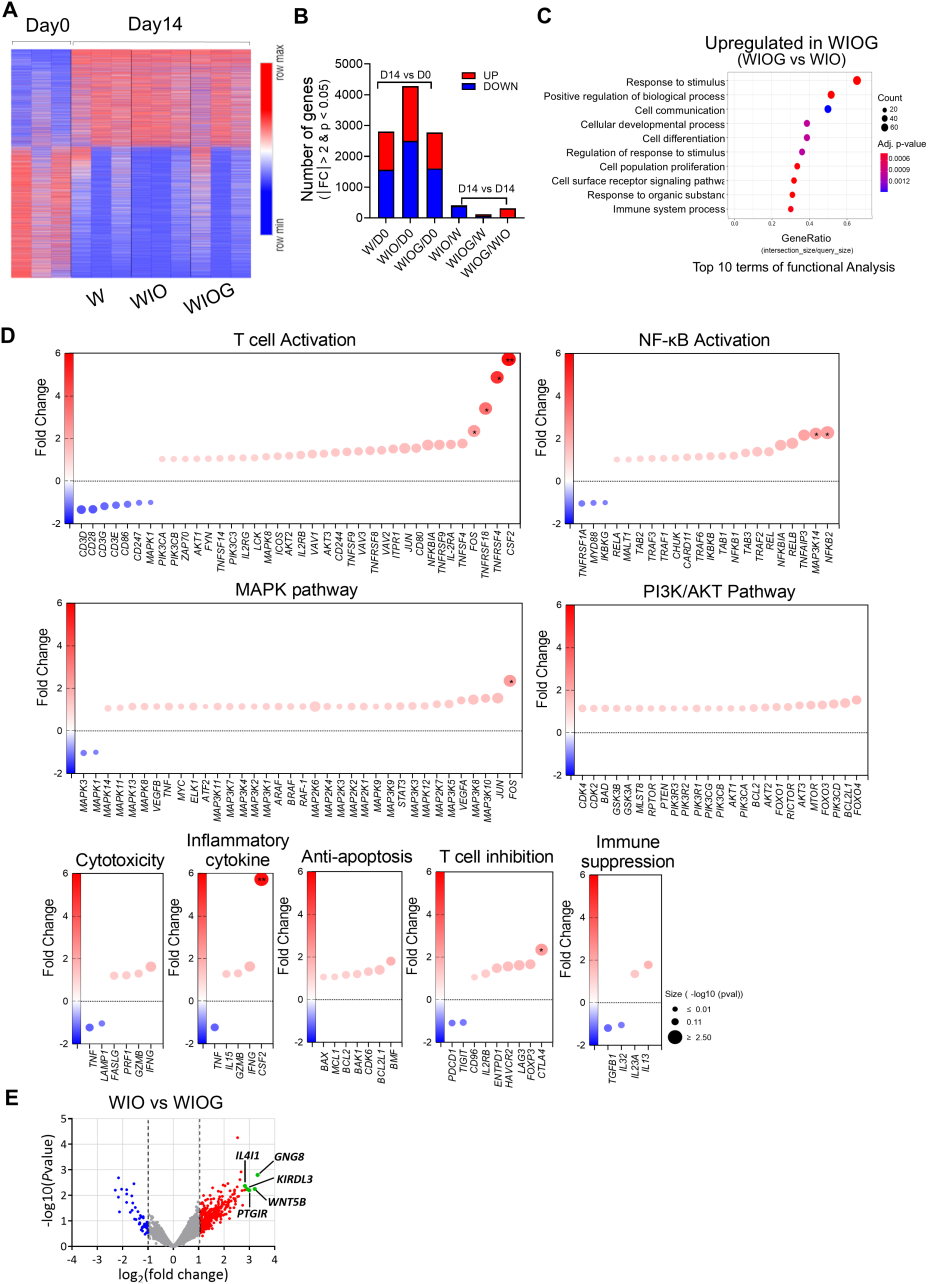
Gene expression signature of TILs after expansion. RNA was isolated from FACS-sorted CD8^+^ T cells from the W, WIO, and WIOG groups, followed by RNA sequencing, bioinformatics analysis, and differential gene expression **(DEG)** analysis. **(A)** The heatmap depicts the clustering of 4,975 differentially expressed genes on Day 14 compared to Day 0 (|fc|≥2 & raw. *P* < 0.05). Warm colors (red) indicate upregulated genes, whereas cool colors (blue) indicate downregulated genes relative to the average expression in Day 0 cells. **(B)** Number of total DEGs and the number of upregulated or downregulated genes identified between Day 14 and Day 0 in each condition, as well as between different treatment groups at Day 14. Red bars indicate upregulated genes, while blue bars indicate downregulated genes. **(C)** Gene Ontology Biological Process (GOBP) pathway analysis showing the top 10 pathways upregulated in Day14 WIOG compared to Day 14 WIO. **(D)** Dot plots representing fold changes in gene expression in the Day 14 WIOG group for genes associated with various biological pathways, including T cell activation, NF-κB activation, MAPK pathway, PI3K/AKT pathway, cytotoxicity, inflammatory cytokines, anti-apoptosis, T cell inhibition, and immune suppression. The x-axis indicates the fold change relative to Day 14 WIO. Genes with increased expression appear in warm colors (red), whereas decreased expression is shown in cool colors (blue). Dot size reflects the −log_10_ adjusted P value. **(E)** Volcano plot showing log_2_ fold changes and −log_10_ adjusted P values for gene expression differences between Day 14 WIOG and Day 14 WIO (|fold change| ≥ 2 and adjusted P < 0.05).

Overall, TILs expanded under the WIOG condition consistently exhibited the highest levels of granzyme B expression in both CD8^+^ and CD4^+^ T cells, suggesting enhanced cytotoxic potential under this condition.

### 3.3 Gene expression signature of expanded TILs using RNA sequencing

To investigate molecular changes induced by anti-GITR stimulation, we performed RNA sequencing on CD8^+^ T cells isolated from TILs expanded under three different conditions (W, WIO, and WIOG) for 14 days (**Figure 3**). A total of 4,975 genes were differentially expressed (|fold change| ≥ 2, raw *P* < 0.05) (**Figure 3A**). When compared to Day 0, the number of differentially expressed genes (DEGs) was 2,803 in W, 4,279 in WIO, and 2,774 in WIOG conditions. Direct comparison between WIOG and WIO identified 311 DEGs, of which 278 were upregulated and 33 were downregulated in the WIOG group (**Figure 3B**).

Gene Ontology Biological Process (GOBP) analysis revealed that genes upregulated in WIOG were significantly enriched in pathways related to stimulus response, cell communication, and immune system processes, suggesting enhanced immunological activity and cellular responsiveness (**Figure 3C**).

T cell activation markers, including *CSF2* (2-fold), *TNFRSF4* (3-fold), *TNFRSF18* (4-fold), and *FOS* (5-fold), were significantly elevated in WIOG condition relative to WIO (**Figure 3D**). Additionally, critical regulators of the NF-κB signaling pathway—*NFKB2* (2.3-fold) and *MAP3K14* (2.2-fold)—were also significantly upregulated in WIOG. Genes involved in MAPK and PI3K/Akt signaling cascades, which are known to be potentiated by GITR activation, similarly exhibited increased expression in WIOG (**Figure 3D**).

Furthermore, cytotoxicity-associated genes including *IFNG, GZMB, PRF1, and FASLG* as well as anti-apoptotic genes such as *BMF, BCL2L1, CDK6, BAK1, BCL1, MCL1*, and *BAX*, were enriched in WIOG-expanded TILs, supporting enhanced survival and effector function in this group (**Figure 3D**).

Conversely, several genes involved in T cell inhibition or immunosuppression, such as *TIGIT*, *PDCD1*, *IL32*, and *TGFB1*, were downregulated in the WIOG group compared to WIO, while *CTLA4* and *FOXP3* expression levels were increased (**Figure 3D**).

Among the significantly upregulated genes in the WIOG condition compared to WIO by DEG analysis, *GNG8* (10.00-fold), *WNT5B* (9.28-fold), *PTGIR* (7.98-fold), *KIR3DL3* (7.51-fold), and *IL4I1* (7.08-fold) showed the highest fold changes, representing the most strongly induced transcripts (**Figure 3E**).

Collectively, these findings indicate that the WIOG condition induces a robust and functionally diverse transcriptional alteration in TILs, which may contribute to enhanced anti-tumor activity.

### 3.4 Anti-GITR enhanced the cytotoxicity of expanded TILs against autologous primary cancer cells

**Figure 4:**
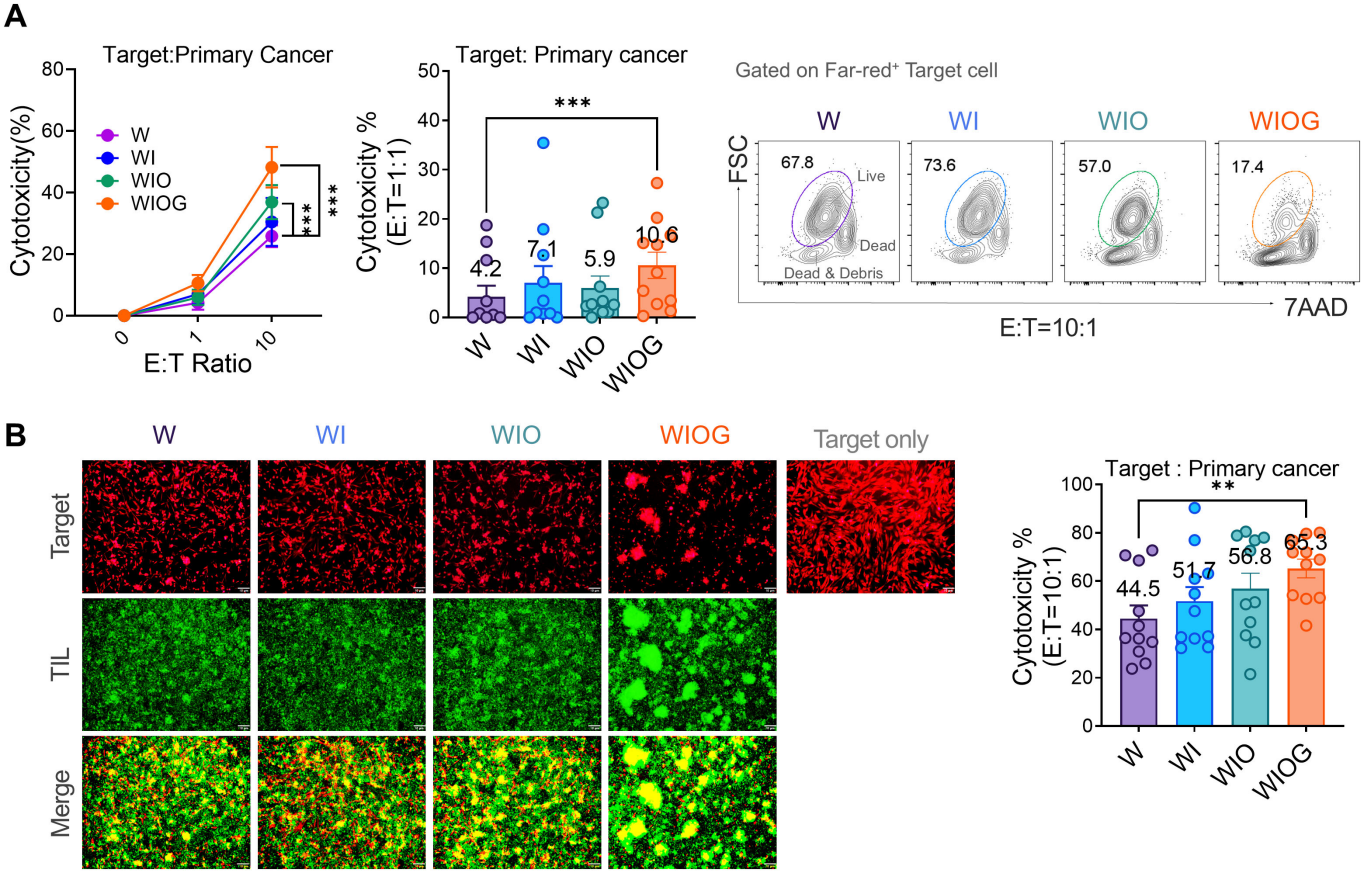
Cytotoxicity of expanded TILs against autologous primary cancer cells. **(A)** Left: Cytotoxic activity of TILs cultured under four different expansion conditions (W, WI, WIO, and WIOG) was assessed against autologous primary cancer cells using the Far Red/7-AAD flow cytometry assay at various effector-to-target (E:T) ratios. Middle: Bar graph showing the percentage of cytotoxicity at an E:T ratio of 1:1 for each condition. Right: Representative plot of 7AAD^-^ Live cells (gated on the Far-red^+^ target cell population) under each expansion condition at an E:T ratio of 10:1. **(B)** Fluorescence microscopy analysis of TIL cytotoxicity against primary cancer cells. Left: Representative images of target cells labeled with Far Red (red) and expanded TILs labeled with Calcein (green) under four different expansion conditions, acquired at E:T ratio of 10:1. Bottom panels show merged images of target cells and TILs. Scale bar, 10 μm. Right: Quantification of TIL cytotoxicity against autologous primary cancer cells at an E:T ratio of 10:1. Data are presented as mean ± SD (n=10). Statistical analyses were performed using the Wilcoxon-test (**P < 0.01, ***P < 0.001).

To evaluate the functional capability of expanded TILs, we assessed the *in vitro* cytotoxicity against autologous primary cancer cells. At an effector-to-target (E:T) ratio of 10:1, both the WIO and WIOG groups exhibited significantly enhanced cytolytic activity compared to the W group (36.9% and 48.9% vs. 17.3%, *P* = 0.0009 for both; **Figure 4A**). Moreover, the WIOG group demonstrated significantly higher cytotoxic activity than all other groups, particularly at the lower E:T ratio of 1:1 (**Figure 4A**). Consistent results were observed when targeting K562 and A2780cis cell lines (**Supplementary Figures 4A, B**).

Furthermore, fluorescence microscopy–based cytotoxicity assays demonstrated significantly greater target cell lysis in the WIOG group (65.3%) compared to the W group (44.5%, *P* = 0.0018; **Figure 4B**).

Collectively, these findings indicate that anti-GITR stimulation augments TIL-mediated cytotoxicity against primary cancer cells, potentially contributing to improved antitumor efficacy.

### 3.5 Expanded TIL with anti-GITR showed *in vivo* anti-tumor effect and persistence

**Figure 5:**
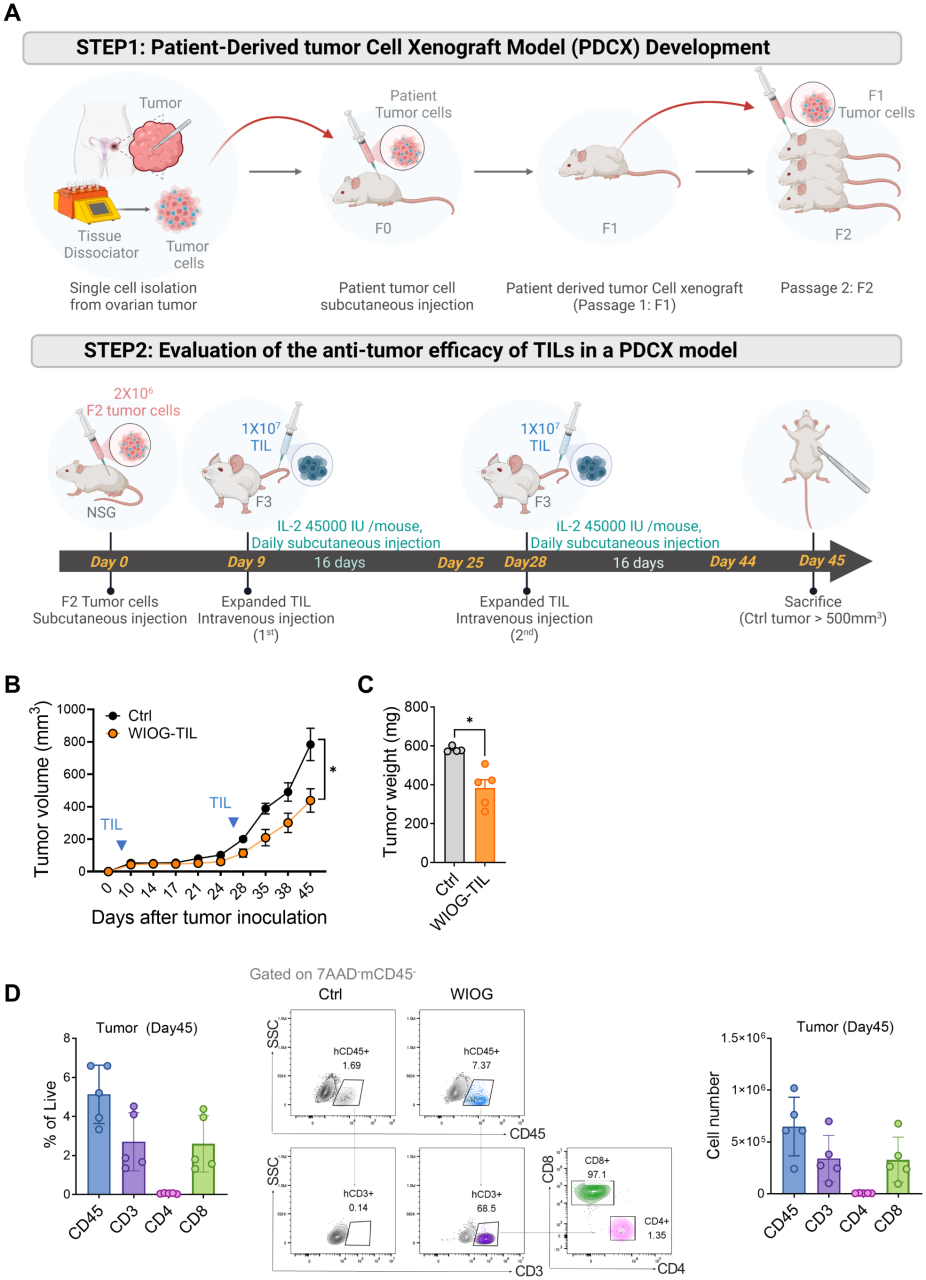
*In vivo* therapeutic effects of expanded TILs in the PDCX model. **(A)** Schematic illustration of the development and treatment protocol of the patient-derived tumor cell xenograft (PDCX) model. Upper panel: Ovarian tumor tissues were dissociated into single cells and subcutaneously injected into NSG mice to establish F0 tumors, which were serially passaged to generate F1 and F2 tumors. Lower panel: For therapeutic evaluation, F2 tumor cells were subcutaneously inoculated into NSG mice on Day 0. Expanded TILs (1 × 10^7^) were administered intravenously on Day 9 and Day 28. IL-2 (45,000 IU/mouse) was injected subcutaneously daily for 16 days following each TIL infusion (n = 5 per each group). **(B)** Tumor volumes were measured at the indicated time points following tumor inoculation. **(C)** Tumor weights were measured at the time of sacrifice. Data are presented as mean ± SD (n = 5 per group). **(D)** TIL infiltration in tumors was assessed by flow cytometry on Day 45. Left: Percentages of human CD45^+^, CD3^+^, CD4^+^, and CD8^+^ T cells in tumor tissues. Middle: Representative dot plots showing human CD45^+^, CD3^+^, CD4^+^, and CD8^+^ T cells gated on 7AAD^-^mCD45^-^ cells in tumors. Right: Absolute numbers of human CD45^+^, CD3^+^, CD4^+^, and CD8^+^ T cells in tumors from the WIOG-TIL group. Data are presented as mean ± SD (n=5). Statistical analyses were performed using the Mann-Whitney test (*P < 0.05).

To evaluate the therapeutic efficacy of expanded TILs *in vivo*, we established a patient-derived tumor cell xenograft (PDCX) model in NSG mice. Tumor cells (2 × 10^6^) from the F2 generation were subcutaneously injected into NSG mice, followed by intravenous administration of 1 × 10^7^ expanded WIOG TILs on days 9 and 28. The WIOG TILs used for adoptive transfer were obtained following rapid expansion (REP) and exhibited a high proportion of CD3^+^ cells (93%) and a strong cytotoxic profile, with 85.6% of CD8^+^ T cells expressing granzyme B (**Supplementary Figure S6**). To support TIL persistence and activity, mice received daily subcutaneous injections of IL-2 (45,000 IU/mouse) (**Figure 5A**).

Consistent with the *in vitro* findings, anti-GITR–expanded TILs significantly inhibited tumor growth by 56% at day 45 compared to the control group receiving IL-2 alone (**Figure 5B**). Tumor weights at the endpoint were also significantly reduced in the WIOG-treated group (383.20 ± 97.47 mg) relative to the control group (581.50 ± 14.48 mg) (**Figure 5C**).

To evaluate the *in vivo* persistence and tumor infiltration of adoptively transferred TILs, tumors were collected at the experimental endpoint (day 45), enzymatically dissociated into single-cell suspensions, and analyzed by flow cytometry. In the TIL-treated group, human CD45^+^ cells comprised 5.13 ± 1.49% of total live cells within the tumor. Among this population, CD3^+^, CD4^+^, and CD8^+^ T cells accounted for 2.70 ± 1.49%, 0.06 ± 0.02%, and 2.60 ± 1.45%, respectively (**Figure 5D**). The absolute number of tumor-infiltrating CD3^+^ T cells was approximately 3.40 × 10^5^, the majority of which were CD8^+^ T cells (approximately 3.30 × 10^5^, 96.35%). The proportion of CD8^+^ cells within the CD3^+^ population increased in all organs *in vivo*, ranging from 91.42% in the lung to 96.35% in the tumor, compared to pre-injection TILs (67.85%) (**Supplementary Figure S6A**). These findings suggest that CD8^+^ T cells expanded more efficiently than CD4^+^ T cells following adoptive transfer. The absolute numbers of infiltrating CD3^+^ TILs in the blood, lung, liver, and spleen were 3.67 × 10^5^, 2.44 × 10^6^, 1.77 × 10^6^, and 2.50 × 10^6^, respectively (**Supplementary Figures 6B,C**).

Collectively, these results indicate that WIOG-expanded TILs not only exert potent anti-tumor effects in the PDCX model but also demonstrate sustained *in vivo* persistence and broad tissue distribution for at least 45 days post-infusion.

## 4 Discussion

ACT using TILs has demonstrated significant therapeutic potential in the treatment of melanoma. However, despite advancements in TIL production protocols and the enrichment of tumor-reactive T cells, clinical outcomes in ovarian cancer (OC) have remained suboptimal. In this study, we established a novel TIL culture protocol aimed at enhancing the therapeutic efficacy of TILs for OC. By incorporating agonistic anti-GITR stimulation alongside IL-2/IL-15/IL-21 and antagonistic anti-PD-1 treatment during the early phase of TIL expansion, we achieved enhanced TIL proliferation, an increased frequency of CD8^+^ T cells, and improved anti-tumor activity against autologous primary OC cells both *in vitro* and *in vivo*.

Cancer immunotherapy targeting GITR has demonstrated potent anti-tumor immune responses and tumor regression in various murine cancer models. Several clinical trials are currently evaluating GITR-targeted therapies, including GITR ligand (GITRL) and agonistic anti-GITR antibodies, in patients with solid tumors (e.g., NCT02697591, NCT01239134, NCT03295942). A previous study has shown that GITR ligation can enhance *ex vivo* TIL proliferation in hepatocellular carcinoma-derived TIL cultures (32). Another study has reported that GITR agonism augmented cellular metabolism, thereby supporting CD8^+^ T cell proliferation in murine models (30). However, the *in vitro* and *in vivo* therapeutic efficacy of GITR-enhanced TILs against autologous cancer cells has not been previously demonstrated.

In fact, the clinical development of GITR agonists has been hindered by limited antitumor efficacy and immune-related toxicities reported in early clinical studies (33, 34). Our approach differs from systemic administration, as GITR agonists are applied only during the ex vivo TIL expansion process, thereby enhancing TIL function while avoiding systemic toxicity. Furthermore, in additional analyses, we confirmed that anti-GITR antibodies were not detectable in the expanded cells after the Rapid Expansion Protocol (REP) (data not shown).

In the present study, to rejuvenate TILs during cultures, we employed an agonistic anti-GITR antibody to enhance CD8^+^ T cell activation and survival while suppressing Tregs. To further overcome T cell exhaustion, a PD-1 inhibitor was added to block the PD-1/PD-L1 axis, thereby providing a synergistic context for anti-GITR efficacy. Our results showed a significantly greater fold expansion of CD8^+^ T cells and an increased CD8^+^ Teff/Treg ratio in the WIOG group (anti-GITR + cytokines + PD-1 blockade) compared to the WIO group (cytokines + PD-1 blockade), although both groups exhibiting increases in total cell numbers and CD8^+^ cell counts. Additionally, GzmB expression and cytotoxic activity against autologous cancer cells were more pronounced in the WIOG group than in the WIO group. Taken together, our study demonstrates that co-targeting GITR and PD-1 during early TIL expansion markedly improves both the proliferative capacity and antitumor functionality of TILs, offering a promising strategy to enhance adoptive cell therapy for ovarian cancer.

At present, we do not have a definitive biomarker to predict which donors benefit the most. However, we hypothesize that donor TILs with higher baseline GITR expression at the start of culture (Day 0) may be more responsive to anti-GITR stimulation, thereby showing greater enhancement under WIOG conditions. To test this hypothesis, we examined the correlation between baseline GITR expression levels on CD3^+^, CD4^+^, and CD8^+^ T cells (D0) and the cytotoxic activity of WIOG-expanded TILs on day 14 (D14) against autologous primary cancer cells. Our analysis revealed that baseline GITR expression on CD3^+^ and CD4^+^ T cells positively correlated with the cytotoxicity of D14 WIOG-expanded TILs (R² = 0.4117, P = 0.0455; R² = 0.4704, P = 0.0286, respectively), whereas CD8^+^ T cells showed only a weak correlation (R² = 0.2032, P = 0.1910). These results suggest that higher baseline GITR expression, particularly on CD3^+^ and CD4^+^ subsets, may serve as a potential predictive biomarker for responsiveness to anti-GITR stimulation during TIL expansion. (**Supplementary Figure S7**).

Understanding the memory phenotype of T cells within TIL populations is essential for optimizing immunotherapeutic strategies. EMRA T cells, a subset of terminally differentiated T cells that re-express CD45RA, are generally considered highly differentiated and are often associated with terminal differentiation or even senescence. These cells often express elevated levels of inhibitory checkpoint molecules such as PD-1, contributing to T cell exhaustion and reduced antitumor efficacy (35, 36). To assess the memory phenotype, we analyzed the expression of CD45RA and CCR7 on CD4^+^ and CD8^+^ T cells. In line with previous reports indicating that ovarian cancer TILs predominantly exhibit an effector memory phenotype (19), our data showed that the majority of CD4^+^ and CD8^+^ T cells in the expanded TIL products were effector memory cells. Although the WIOG group exhibited a slight increase in the frequency of EMRA cells among both CD4^+^ and CD8^+^ populations compared to the W group, these changes were not statistically significant and were lower than those observed in the WIO group. Overall, our findings suggest that the addition of the anti-GITR antibody during the initial phase of TIL expansion does not induce major alterations in the T cell memory phenotype.

Upon activation in response to antigen stimulation, T cells upregulate immune checkpoint molecules such as PD-1 and CTLA-4. Although these molecules regulate the immune response by providing inhibitory signals that prevent excessive T cell activation and autoimmune damage (37, 38), several studies have reported that PD-1^+^CD8^+^ T cells retain functional capacity, including the ability to recognize autologous tumor cells, secrete IFN-γ, and upregulate 4-1BB, as well as containing a fraction of tumor neoantigen-specific T cells (39–41). In our study, CD8^+^ T cells across all groups exhibited modest expression levels of PD1 and CTLA-4, representing highly activated T cells, including tumor-reactive population.

Based on our RNA sequencing analysis, TILs expanded under the WIOG condition exhibited more robust NF-κB pathway activation and greater induction of anti-apoptotic genes compared to the WIO group. Among genes associated with T cell activation, *CSF2, TNFRSF4*, and *TNFRSF18* were significantly upregulated in the WIOG group, potentially reflecting enhanced NF-κB activation (42, 43). Specifically, *TNFRSF4* (OX40) and *TNFRSF18* (GITR) are known to directly promote T cell activation, while *CSF2* (GM-CSF) supports the activation of dendritic cells and macrophages, thereby enhancing antigen presentation and sustaining T cell responses (44, 45). Anti-GITR signaling has been shown to activate NF-κB in T cells through TNF receptor-associated factors, leading to reduced apoptosis and enhanced T-cell survival via the upregulation of anti-apoptotic molecules such as Bcl-xL (46, 47). In addition, NF-κB signaling plays a critical and distinct role in the development and maintenance of CD8^+^ T cell memory (48). In the present study, we also observed significant upregulation of *FOS*, a gene typically induced via the MAPK pathway. FOS plays a key role in T cell proliferation and differentiation, contributing to the development of effector T cell populations (49). Notably, our analysis identified *IL4I1* as one of the most differentially expressed genes in CD8^+^ T cells following GITR stimulation. IL4I1 (interleukin-4-induced-1) is an immunoregulatory enzyme predominantly expressed by macrophages, dendritic cells, and subsets of T cells. While IL4I1 has been traditionally associated with immune suppression, limiting CD8^+^ T cell proliferation and effector functions, recent evidence suggests it may also support rapid expansion of effector CD8^+^ T cells under certain conditions (50). Moreover, elevated IL4I1 expression has been reported as a hallmark of an inflamed tumor microenvironment (TME) and has been correlated with improved responses to immunotherapy (51). These findings suggest that IL4I1 may exert context-dependent dual roles, reflecting a complex interplay between immune activation and suppression. Further investigation will be required to clarify its precise function in our system. Furthermore, we identified a strong induction of *WNT5B* (9.28-fold) in the WIOG group. WNT5B, a member of the WNT family closely related to WNT5A, is involved in regulating cell migration, proliferation, and differentiation. Importantly, WNT signaling has been implicated in promoting the formation of memory T cells in response to tumor antigens, thereby enhancing long-term anti-tumor immunity (52). Taken together, anti-GITR agonist induced molecular alterations related to enhancing T-cell survival, proliferation, and T-cell activation.

FOXP3^+^ CD8^+^ Tregs have been reported to be present in high proportions within the TILs of ovarian cancer patients, where they exert immunosuppressive functions by expressing CTLA-4 and secreting IL-10 (53). The presence of FOXP3^+^ CD8^+^ Tregs is associated with cancer prognosis, with elevated levels potentially correlating with a poorer clinical outcome (54). In our study, the baseline proportion of FOXP3^+^ CD8^+^ Tregs prior to culture was low (0.04 ± 0.08%). After expansion, this population remained low in the WIOG group (0.48 ± 1.28%) compared to the other groups, which showed increases up to 1.16 ± 2.38%.

The process of generating TILs typically involves a two-step expansion protocol, consisting of a pre-rapid expansion phase (pre-REP) followed by a rapid expansion phase (REP). It is generally expected that the cytotoxicity of TILs may decline from the pre-REP to the REP stage due to phenotypic changes and exhaustion caused by repeated stimulation and prolonged culture (55). In this study, we utilized a feeder-free REP protocol to expand TILs for adoptive transfer into xenograft mice. The post-REP TILs achieved a 27-fold expansion, with CD45^+^ and CD3^+^ cells comprising 95% and 93% of the population, respectively (**Supplementary Figure S5**). Importantly, these post-REP TILs retained most of the key features of pre-REP TILs, including a high proportion of GzmB^+^ cells, and demonstrated potent anti-tumor efficacy upon *in vivo* administration.

In addition, our study incorporated *in vitro* and *in vivo* toxicity assessments using primary cancer cells, enabling a closer approximation of the human *in vivo* environment and demonstrated the therapeutic efficacy of GITR-enhanced TILs under clinically relevant conditions (48.9% cytotoxicity *in vitro* and 56% tumor reduction *in vivo*). Notably, our *in vivo* experiments employed a PDCX model (31), which closely mimics the human tumor, providing a highly pertinent platform to evaluate the therapeutic potential of GITR-enhanced TILs in settings representative of human cancer. Collectively, these findings indicate that early anti-GITR stimulation significantly improved the anti-tumor efficacy of TILs, with sustained functional enhancement observed throughout the REP phase and translated into effective tumor control *in vivo*.

We acknowledge that *in vivo* validation was initially performed using TILs from a single representative donor due to ethical and resource constraints associated with large-scale TIL expansion. Validation across multiple donors would undoubtedly strengthen the robustness of our findings; however, such experiments require substantial TIL quantities and are technically challenging within PDX systems. To partially address this limitation, we additionally evaluated WIOG-expanded TILs derived from another donor in an OVCAR3 xenograft model. Although this model does not represent a patient-derived xenograft, anti-GITR–expanded TILs significantly inhibited tumor growth by 56% at day 53 compared to the vehicle (IL-2) control group (**Supplementary Figure S8**). These complementary data further support the *in vivo* relevance and reproducibility of GITR-enhanced TIL efficacy across different donor background.

In conclusion, we demonstrated that incorporating an agonistic anti-GITR antibody into the initial TIL culture significantly enhances TIL proliferation, increases the proportion of CD8^+^ T cells and GzmB^+^ CD8^+^ effector cells, and improves anti-tumor efficacies both *in vitro* and *in vivo*. Furthermore, we provided a comprehensive characterization of the genetic, phenotypic, and functional profiles of ex vivo–expanded ovarian TILs enhanced by anti-GITR stimulation. These findings suggest that TIL therapy incorporating anti-GITR agonism may represent a promising strategy to improve clinical outcomes in ovarian cancer patients.

## Data Availability

The datasets presented in this study can be found in online repositories. The names of the repository/repositories and accession number(s) can be found below: https://www.ncbi.nlm.nih.gov/geo/, GSE278940.
