# Supplementary material for: Enhanced anti-tumor efficacy of tumor-infiltrating lymphocytes by GITR agonist in ovarian cancer
Source: Front Immunol. 2025 Nov 6;16:1670841. doi: 10.3389/fimmu.2025.1670841 (PMC12631380; doi:10.3389/fimmu.2025.1670841)
Supplement: Supplementary file 1 [file Supplementaryfile1.docx]

Supplementary Material

# Supplementary Figures


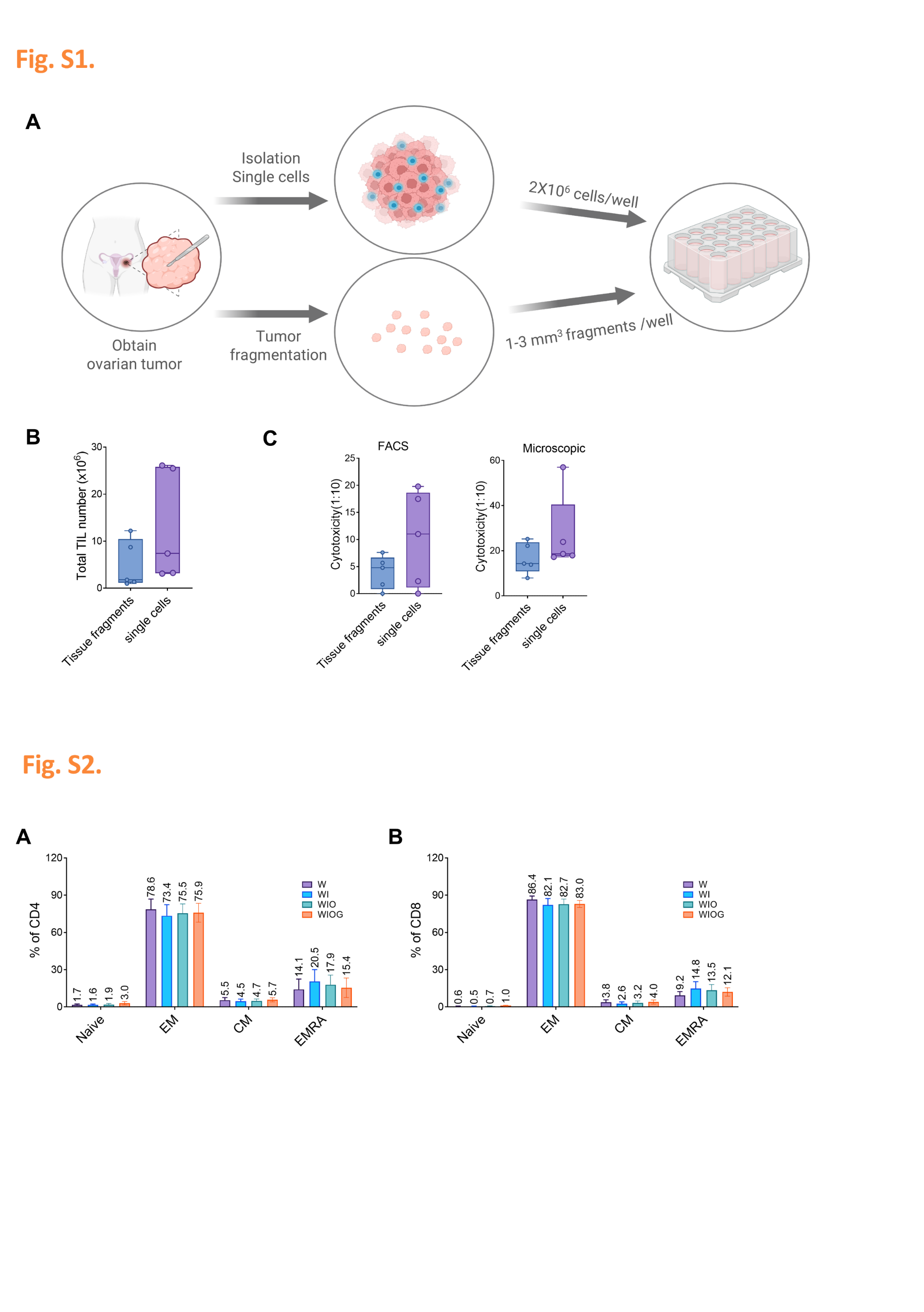


**Supplementary Figure 1. Isolation and characterization of ovarian tumor cells.**

**(A)** Ovarian tumors were collected and processed to either isolate single cells or generate small tissue fragments (1-3 mm³). Single cells were seeded at a density of 2×10⁵ cells per well, while tissue fragments were seeded at 1-3 mm³ per well. TILs were then expanded with IL-2 for 14 days. **(B)** Total number of expanded TILs from tumor fragments and single cells. The mean number of total cells in TILs derived from tumor fragments was 5.01 ± 5.14 × 10^6^ cells and those from single cells was 13.07 ± 11.75× 10^6^. **(C)** Cytotoxicity of TILs against autologous cancer cells was analyzed using the 7-AAD/Far-red assay by FACS and Far-red staining, microscopically with the indicated E:T ratio.

**Supplementary Figure 2. CD4+ or CD8+ Tregs in expanded TIL**

Percentages of CD4^+^ Treg and CD8^+^ Treg cells were analyzed using flow cytometry. Data are presented as mean ± SD (n=10). Statistical analysis was performed using the Wilcoxon-test (**P* < 0.05, ** *P* < 0.01, *** *P* < 0.001)

**
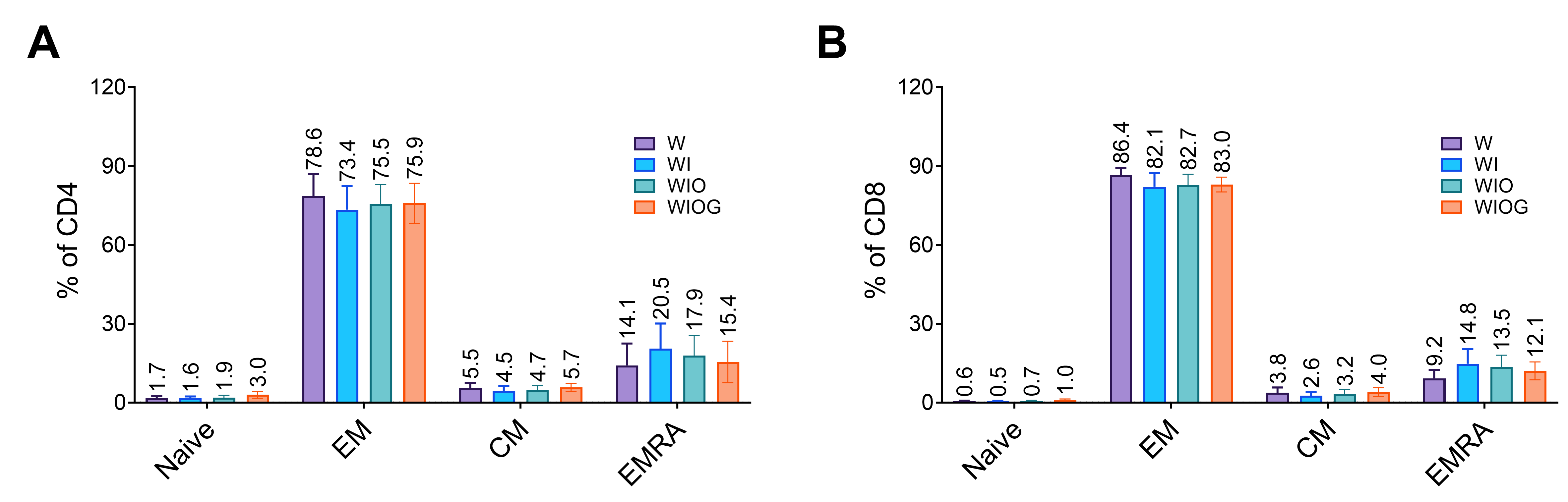
 Supplementary Figure 3. Naive/memory phenotype of expanded TIL**

Proportion of naïve, T CM, T EM and T EMRA subsets in CD4^+^ **(A)** and CD8^+^ T cells **(B)** of expanded TILs were analyzed using flow cytometry.


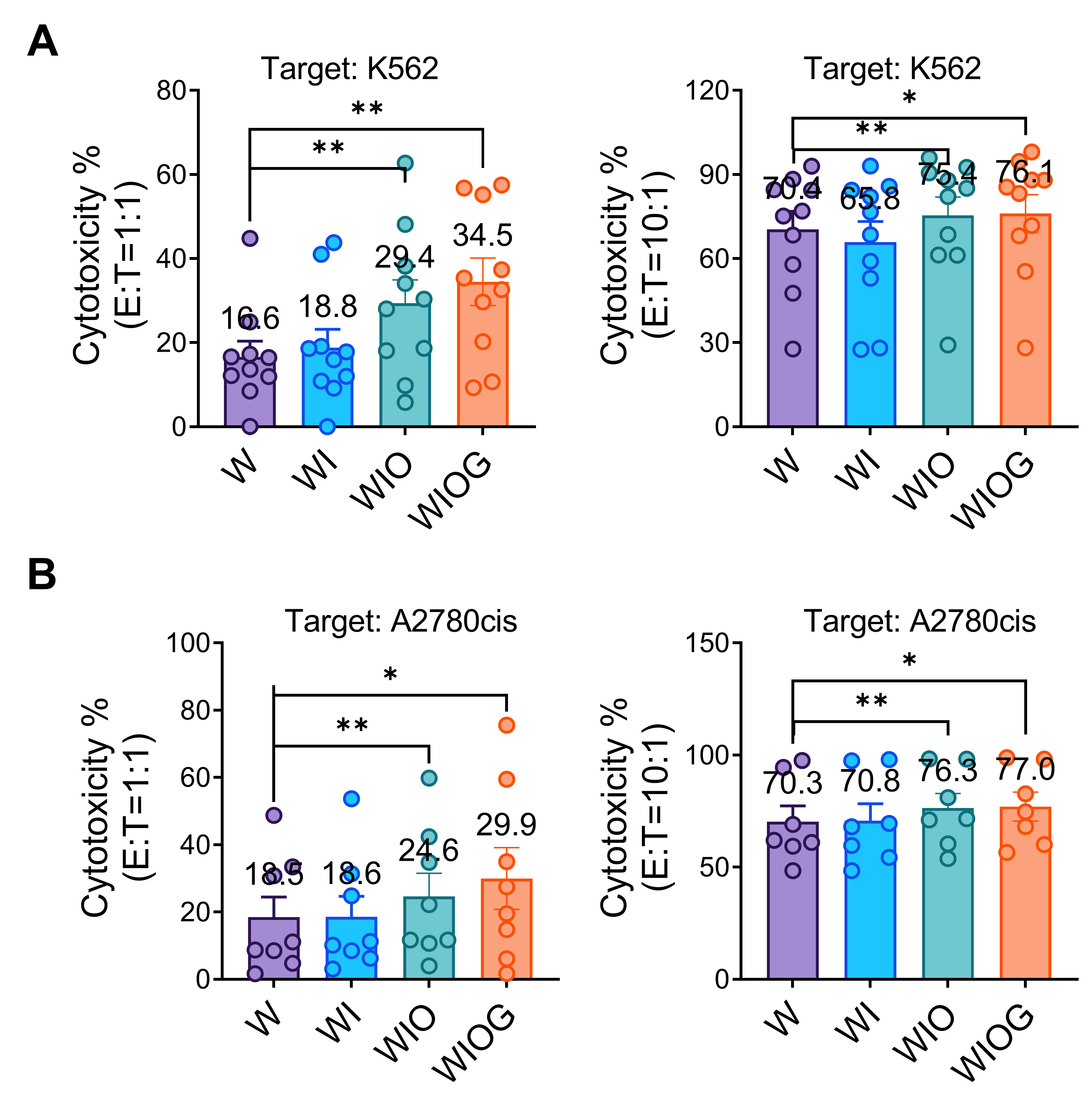


**Supplementary Figure 4. Cytotoxicity of Expanded TILs against cancer cell lines**

Cytotoxicity of Expanded TILs in four different expansion conditions against K562 cancer cell line **(A)** and A2780cis ovarian cancer cell line **(B)** was analyzed using the CFSE/7-AAD assay with the indicated E: T ratio Data are presented as mean ± SD (n=10). Statistical analysis was performed using Wilcoxon-test (*p < 0.05, **p < 0.01)


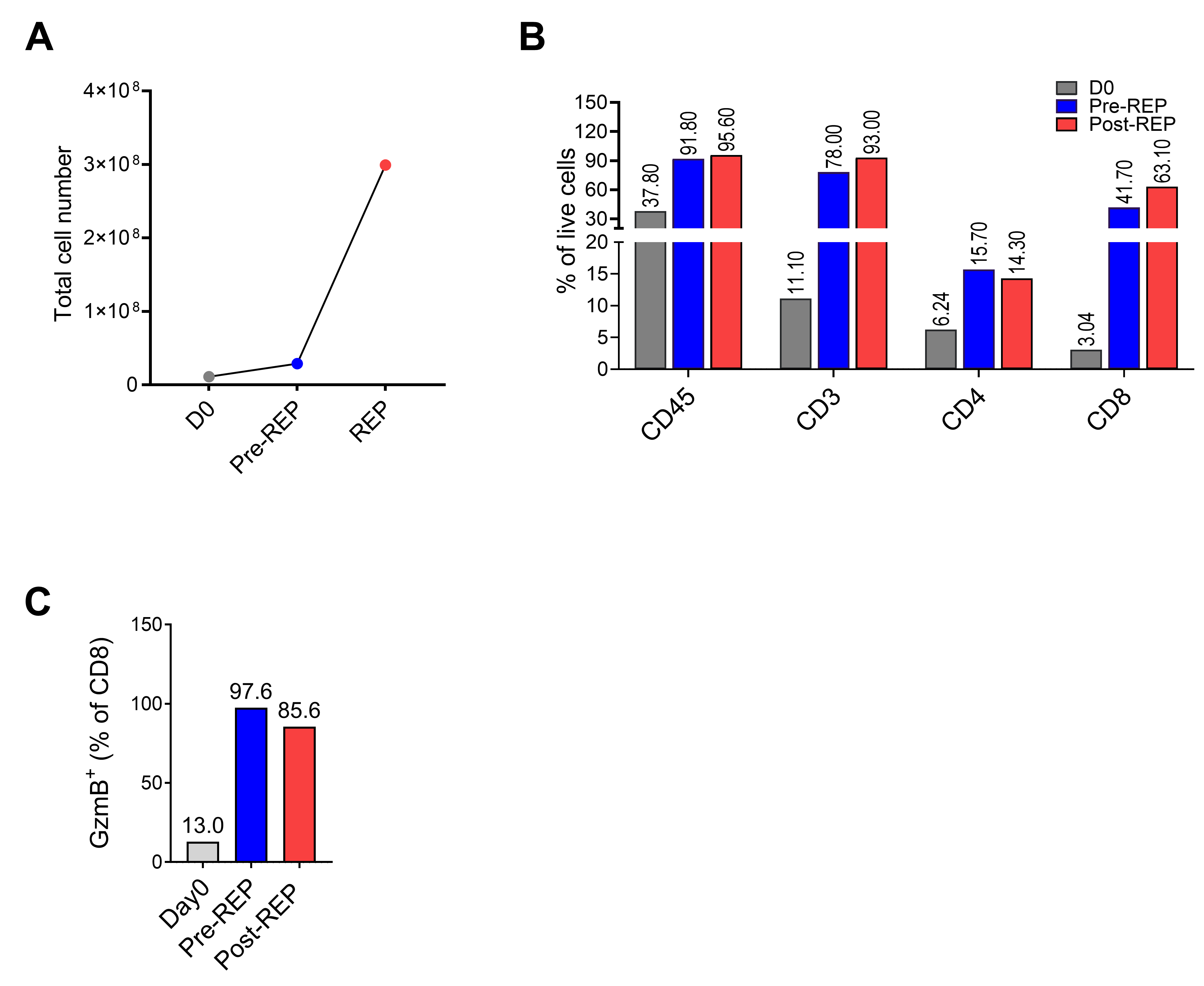


**Supplementary Figure 5. Characterization of immune cells before and after REP**

**(A)**Total cell number at different stages: Day 0, Pre-REP, and Post-REP. **(B)** Percentages of CD45^+^, CD3^+^, CD4^+^, and CD8^+^ T cells at D0, Pre-REP, and Post-REP were analyzed using flow cytometry. **(C)** Percentage of CD8^+^ T cells expressing GzmB at D0, Pre-REP, and Post-REP was analyzed using flow cytometry.


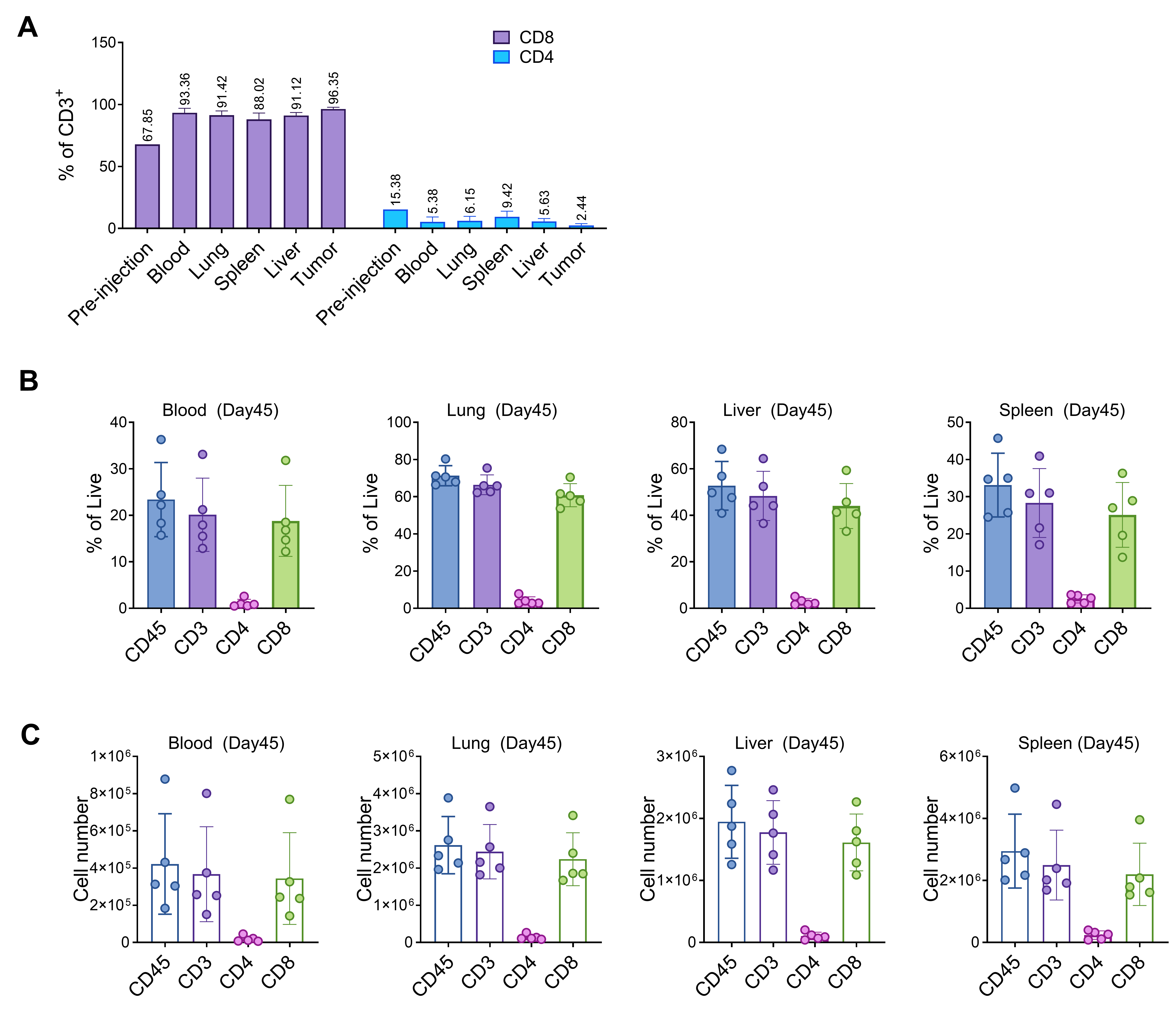


**Supplementary Figure 6. Analysis of injected TIL infiltration in tissues of PDCX model on day 45**

The percentage and absolute number of intravenously injected WIOG TILs in blood, lung, liver, spleen was measured by FACS after the mouse was sacrificed. **(A)** Percentages of human CD8^+^ T cells and CD4^+^ T cells within CD3+ cells in the blood, lung, liver, and spleen of the WIOG group were analyzed. **(B)** Percentages of human CD45^+^, CD3^+^, CD4^+^, and CD8^+^ T cells in in blood, lung, liver and spleen of WIOG group were analyzed. **(C)** Absolute cell number of human CD45^+^, CD3^+^, CD4^+^ , and CD8^+^ T cells in blood, lung, liver and spleen of WIOG group

**
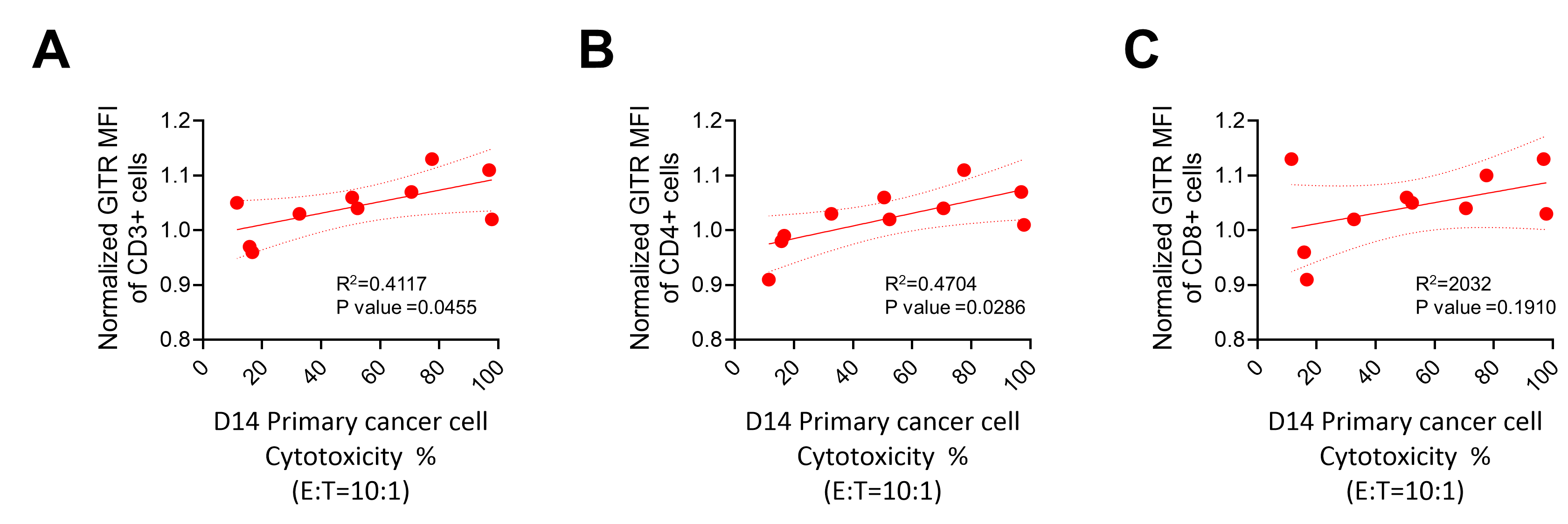
**

**Supplementary Figure 7. Correlation between baseline GITR expression and cytotoxicity of WIOG-expanded TILs.**

Correlation analysis between the normalized mean fluorescence intensity (MFI) of GITR expression on **(A)** CD3⁺, **(B)** CD4⁺, and **(C)** CD8⁺ T cells at day 0 (D0) and the cytotoxicity (%) of WIOG-expanded TILs at day 14 (D14) against autologous primary ovarian cancer cells (E:T = 10:1). GITR MFI values at D0 were normalized to their corresponding isotype control values. Each dot represents an individual donor sample.

**
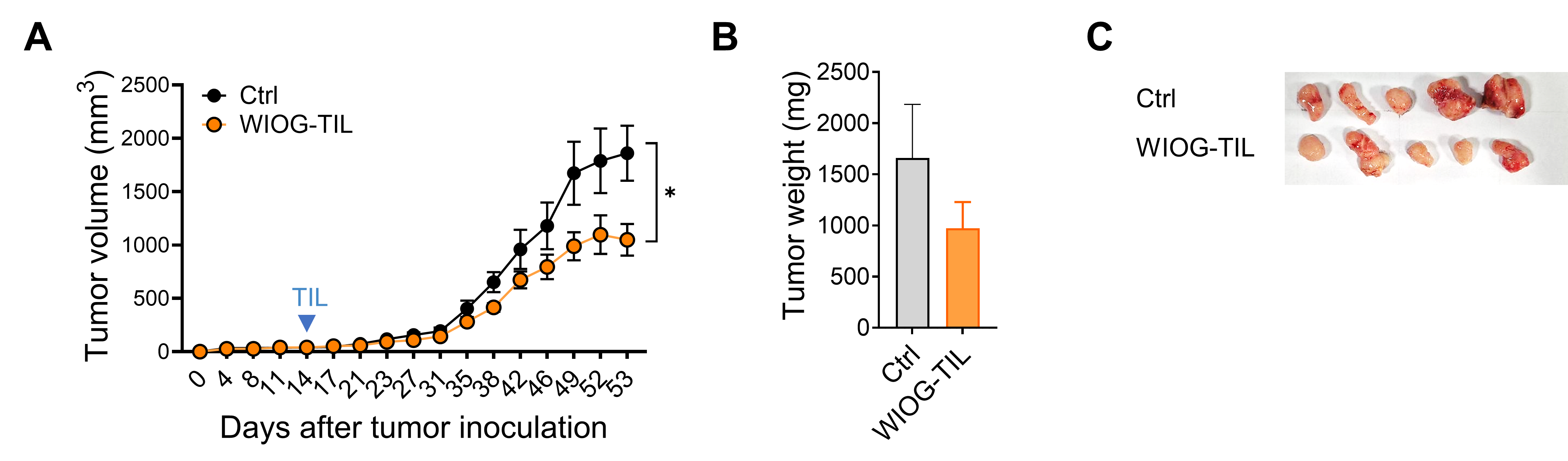
**

**Supplementary Figure 8. In vivo effects of expanded TILs in OVCAR3 xenograft model**

Expanded TILs (1 × 10⁷, WIOG) or vehicle control (Ctrl) were administered intravenously on day 14. Recombinant human IL-2 (45,000 IU per mouse) was injected subcutaneously daily for 2 weeks following each TIL infusion (n = 5 per group). **(A)** Tumor volumes were measured at the indicated time points after tumor inoculation. **(B)** Tumor weights were assessed at the experimental endpoint (day 53). **(C)** Representative images of excised OVCAR3 tumors from each group at the endpoint.
Data are presented as mean ± SD (n = 5 per group).

# Supplementary Tables

**Supplementary Table 1. Patient’s clinical and pathological characteristics**

| No | Patient No | Histopathological subtype | Age | Stage | Tumor tissue weight (g) | Single cell yield from tumor (10^6) |
| --- | --- | --- | --- | --- | --- | --- |
| 1 | SC199 | High-grade Serous Carcinoma, Ovarian | 50s | 2 | 8.8 | 3100 |
| 2 | SC210 | High-grade Serous Carcinoma, Ovarian | 50s | 3 | 3.58 | 390 |
| 3 | SC222 | High-grade Serous Carcinoma, Ovarian | 70s | 3 | 2 | 16 |
| 4 | SC238 | High-grade Serous Carcinoma, Ovarian | 60s | 3 | 13.7 | 150 |
| 5 | SC236 | High-grade Serous Carcinoma, Ovarian | 50s | 3 | 14 | 120 |
| 6 | SC243 | High-grade Serous Carcinoma, Ovarian | 40s | 3 | 6.8 | 220 |
| 7 | SC245 | Parametrial mass of High-grade Serous Carcinoma, Ovarian | 40s | 3 | 21.4 | 268 |
| 8 | SC246 | High-grade Serous Carcinoma, Ovarian | 50s | 3 | 19.7 | 444 |
| 9 | SC248 | High-grade Serous Carcinoma, Ovarian with sarcomatoid component (< 5%) | 50s | 4 | 17.5 | 120 |
| 10 | SC250 | High-grade Serous Carcinoma, Ovarian | 60s | 3 | 13 | 196 |

**Supplementary Table 2. List of used reagents for TIL culture**

| Product | Working concentration | Cat.No | Supplier |
| --- | --- | --- | --- |
| Recombinant human IL-2 | 6000 U/ml or 3000 U/ml | 200-02 | PEPROTECH |
| Recombinant human IL-15 | 180 U/ml | 20-15 | PEPROTECH |
| Recombinant human IL-21 | 40 U/ml | 200-21 | PEPROTECH |
| Human CD3/CD28 T Cell Activator | 10 μg/ml | 10990 | stemcell |
| Anti-human PD-1 antibody | 10 μg/ml | J116 | BioXcell |
| Anti-GITR Agonist antibody | 20 μg/ml | 79053-2 | BPS Bioscience |
| Anti-CD3/CD28/CD2 T Cell Activator | 1×10^6^ /25μL | 10990 | STEMCELL Technologies |

**Supplementary Table 3. List of used antibodies for FACS**

| Antibodies | Fluorescence | Clone | Cat.No | Supplier |
| --- | --- | --- | --- | --- |
| Antibodies for surface staining | | | | |
| anti-mouse IgG1 kappa isotype | FITC | MOPC-21 | 400108 | Biolegend |
| anti-mouse IgG2a kappa isotype | FITC | MOPC-173 | 4002010 | Biolegend |
| anti-mouse IgG1 kappa isotype | PE | MOPC-21 | 555749 | BD Parmingen™ |
| anti-mouse IgG2a kappa isotype | PE | eBM2a | 12-4724-82 | Invitrogen |
| anti-mouse IgG1 kappa isotype | APC | MOPC-21 | 555751 | BD Parmingen™ |
| anti-mouse IgG1 kappa isotype | e450 | eBM2a | 48-4724-82 | Invitrogen |
| anti-mouse IgG1 kappa isotype | AF700 | MOPC-21 | 400144 | Biolegend |
| anti-Rat IgG2a kappa isotype | AF700 | eBR2a | 56-4321-80 | Invitrogen |
| anti-mouse IgG1 kappa isotype | APC/Cyanine7 | MOPC-21 | 400127 | Biolegend |
| anti-mouse IgG2a kappa isotype | BV605 | MOPC-173 | 400270 | Biolegend |
| anti-mouse IgG2b kappa isotype | BV421 | MPC-11 | 400342 | Biolegend |
| anti-mouse IgG1 kappa isotype | BV500 | X40 | 560787 | BD Parmingen™ |
| anti-mouse IgG1 kappa isotype | AF700 | MOPC-21 | 400144 | Biolegend |
| anti-CD45 | FITC | HI30 | 304006 | Biolegend |
| anti-CD3 | eFluor 450 | UCHT1 | 48-0038-42 | eBioscience |
| anti-CD4 | PE | RPA-T4 | 555347 | BD Parmingen™ |
| anti-CD4 | AF700 | RPA-T4 | 557922 | BD Parmingen™ |
| anti-CD8 | APC | RPA-T8 | 555369 | BD Parmingen™ |
| anti-CD56 | APC/Cyanine7 | 5.1H11 | 362512 | Biolegend |
| anti-CCR7 | BV605 | G043H7 | 353224 | Biolegend |
| anti-CD45RA | BV421 | HI100 | 304130 | Biolegend |
| anti-CD137 | AF700 | 4B4-1 | 309816 | Biolegend |
| anti-OX40 | APC/Cyanine7 | Ber-ACT35 (ACT35) | 350022 | Biolegend |
| Anti-CD19 | FITC | HIB19 | 555412 | BD Parmingen™ |
| Anti-CD14 | PE | M5E2 | 555398 | BD Parmingen™ |
| anti-GITR | PE | 621 | 311603 | Biolegend |
| Antibodies for Intracellular staining | | | | |
| anti-FOXP3 | AF700 | PCH101 | 56-4776-41 | Invitrogen |
| anti-Granzyme B | V510 | GB11 | 563388 | BD Parmingen™ |

**Supplementary Table 4. List of used antibodies for FACS to analysis immune cells in vivo study**

| Antibodies | Fluorescence | Clone | Cat.No | Supplier |
| --- | --- | --- | --- | --- |
| Antibodies for surface staining | | | | |
| Anti-mouse CD45 | PE-Cyanine7 | 30-F11 | 25-0451-82 | Invitrogen |
| Anti-human CD45 | V500 | HI30 | 560777 | BD Parmingen™ |
| Anti-human CD3 | PE | HIT3a | 12-0039-42 | Invitrogen |
| Anti-human CD4 | AF700 | RPA-T4 | 557922 | BD Parmingen™ |
| Anti-human CD8 | APC | RPA-T8 | 555369 | BD Parmingen™ |
