# Supplementary figures and images for: Enhanced anti-tumor efficacy of tumor-infiltrating lymphocytes by GITR agonist in ovarian cancer
Source: Front Immunol. 2025 Nov 6;16:1670841. doi: 10.3389/fimmu.2025.1670841 (PMC12631380; doi:10.3389/fimmu.2025.1670841)

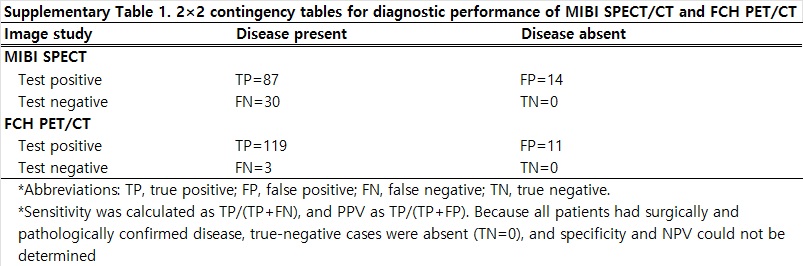

Supplement: Supplementary file 2 [file Supplementaryfile1.jpg]
